# Secondary Data Analyses of Subjective Outcome Evaluation Data Based on Nine Databases

**DOI:** 10.1100/2012/346369

**Published:** 2012-05-22

**Authors:** Daniel T. L. Shek

**Affiliations:** ^1^Department of Applied Social Sciences, The Hong Kong Polytechnic University, Hong Kong; ^2^Public Policy Research Institute, The Hong Kong Polytechnic University, Hong Kong; ^3^Department of Social Work, East China Normal University, Shanghai 200062, China; ^4^Kiang Wu Nursing College of Macau, Macau; ^5^Division of Adolescent Medicine, Department of Pediatrics, Kentucky Children's Hospital, University of Kentucky College of Medicine, Lexington, KY 40536, USA

## Abstract

The purpose of this study was to evaluate the effectiveness of the Tier 1 Program of the Project P.A.T.H.S. (Positive Adolescent Training through Holistic Social Programmes) in Hong Kong by analyzing 1,327 school-based program reports submitted by program implementers. In each report, program implementers were invited to write down five conclusions based on an integration of the subjective outcome evaluation data collected from the program participants and program implementers. Secondary data analyses were carried out by aggregating nine databases, with 14,390 meaningful units extracted from 6,618 conclusions. Results showed that most of the conclusions were positive in nature. The findings generally showed that the workers perceived the program and program implementers to be positive, and they also pointed out that the program could promote holistic development of the program participants in societal, familial, interpersonal, and personal aspects. However, difficulties encountered during program implementation (2.15%) and recommendations for improvement were also reported (16.26%). In conjunction with the evaluation findings based on other strategies, the present study suggests that the Tier 1 Program of the Project P.A.T.H.S. is beneficial to the holistic development of the program participants.

## 1. Introduction

Although the prevention science approach that focuses on risk and protective factors of high-risk adolescent behavior has generated much research and prevention programs in the past few decades, it has been criticized as focusing too much on adolescent problems and pathology. As such, there is an alternative approach that emphasizes the importance of positive youth development. Damon [1] stated that the field of positive youth development (PYD) focuses on each child's talents, strengths, interests, and future potential in contrast to approaches that focus on problems that some youth display when they grow up, such as delinquency and substance abuse.

There are many positive youth development programs in the field. In a review of existing programs on positive youth development, Catalano et al. [2] reviewed 77 programs and concluded that there were 25 successful programs involving 15 positive youth development constructs. These constructs include promotion of bonding, cultivation of resilience, promotion of social competence, promotion of emotional competence, promotion of cognitive competence, promotion of behavioral competence, promotion of moral competence, cultivation of self-determination, promotion of spirituality, development of self-efficacy, development of a clear and positive identity, promotion of beliefs in the future, provision of recognition for positive behavior, provision of opportunities for prosocial involvement, and fostering prosocial norms. Obviously, these positive youth development constructs can be utilized in youth development programs that aim to promote the holistic development of adolescents.

With reference to the intensification of adolescent developmental problems in Hong Kong [3, 4], there are very few systematic and multiyear positive youth development programs in Hong Kong. The existing youth enhancement programs commonly deal with isolated problems and issues in adolescent development (i.e., deficits-oriented programs), and they are relatively short term in nature. To promote holistic development among adolescents in Hong Kong, the Hong Kong Jockey Club Charities Trust initiated and launched a project entitled “P.A.T.H.S. to Adulthood: A Jockey Club Youth Enhancement Scheme” with an earmarked grant of HK$400 million for the initial phase. (P.A.T.H.S. stands for Positive Adolescent Training through Holistic Social Programmes.) Because of the overall success of the initial phase, an additional grant of HK$350 million was earmarked for the extension phase of the project.

There are two tiers of programs (Tier 1 and Tier 2 Programs) in this project. The Tier 1 Program is a universal positive youth development program in which students in Secondary 1 to 3 participate, normally with 20 h of training in the school year at each grade. Because research findings suggest that roughly one-fifth of adolescents would need help of a deeper nature, the Tier 2 Program is generally provided for at least one-fifth of the students who have greater psychosocial needs at each grade (i.e., selective program). To date, more than 244 schools (with 669 schools in the Secondary 1 level, 443 in the Secondary 2 level, and 215 in the Secondary 3 level) and 223,101 students have participated in the Tier 1 Program of the project [5, 6].

Several evaluation strategies have been utilized to evaluate the Project P.A.T.H.S. in Hong Kong. These include objective outcome evaluation, subjective outcome evaluation, qualitative evaluation, process evaluation, and evaluation based on personal construct psychology. Among these evaluation strategies, the subjective outcome evaluation method was used to assess the perceptions of the participants as well as program implementers regarding the program, instructors, and benefits of the program [7, 8]. It is noteworthy that although subjective outcome evaluation or the client satisfaction approach is commonly used in human services to collect the views of the program participants, there are comparatively fewer attempts to carry out subjective outcome evaluation among program implementers [7, 8].

There are several reasons why program implementers should be engaged in the evaluation process. First, by engaging the program implementers in the evaluation process, a more complete picture of the effectiveness of the program can be constructed. In particular, by adding the perspective of the program implementers, bias due to subjectivity of the program participants can be reduced and the related data can enrich our understanding of the program effect. Second, engagement of program implementers is commonly emphasized in different evaluation models. For example, in the utilization-focused evaluation paradigm, it is argued that as different stakeholders are involved in the evaluation process, program implementers' views are legitimately covered [9]. Similarly, based on the standards of the Joint Committee on Standards for Educational Evaluation [10], identification of the stakeholders (Utility Standard 1) involving complete and fair assessment (Proprietary Standard 5) is important. According to these standards, program implementers' views and assessments should be taken into account. Different researchers have also emphasized the importance of engaging different stakeholders in the evaluation process [11–13]. For example, Brandon et al. [14] pointed out that participation of program stakeholders in evaluations improves the relevance and validity of evaluation results.

In the Project P.A.T.H.S., subjective outcome evaluation is used to capture the views of the program participants and implementers. Based on these data, implementers in each school are required to submit a report documenting the effects of the program, including five conclusions that they would like to put down in the report. By utilizing and integrating the five conclusions drawn in the school-based evaluation reports prepared by the program implementers based on the views of both program participants and implementers, the present study conducted secondary data analyses to evaluate the effectiveness of the Tier 1 Program of the P.A.T.H.S. Project. According to Royse [15], secondary data analysis “involves analysis of an existing data set that results in knowledge, interpretations, and conclusions beyond those stated in the original study” (page 201), and it is a kind of unobtrusive research method, which does not need to have direct interaction with the subjects. Studies utilizing secondary data analyses are common in the social science literature [16, 17].

Several studies have been carried out to examine the five conclusions drawn in different cohorts of the Project P.A.T.H.S. [18–20]. Generally speaking, the findings showed that different stakeholders had positive perceptions of the program, instructors, and benefits of the program. There were also suggestions for improvement in the reports. As there are nine databases containing data on the five conclusions, it is exciting to look at the aggregated picture based on secondary data analysis of the available data. As such, the present study was carried out to examine the effectiveness of the Tier 1 Program of the Project P.A.T.H.S. based on the secondary data analyses of conclusions drawn by the program implementers based on the program participants and implementers.

## 2. Methods

### 2.1. Dataset for Secondary Data Analyses

In each year of the Experimental and Full Implementation Phases, after completion of the Tier 1 Program, students and program implementers were invited to respond to subjective outcome evaluation forms (Forms A and B, resp.). The program implementers then prepared a report based on the subjective outcome evaluation data to report the program effectiveness. Throughout the years, a total of 1,327 reports involving 244 schools, 223,101 students, and 9,915 program implementers were collected (Table 1).

There are several parts in Form A:

- participants' perceptions of the program, such as program objectives, design, classroom atmosphere, interaction among the students, and the respondents' participation during class (10 items),
- participants' perceptions of the instructors, such as the preparation, professional attitude, involvement, and interaction with the students (10 items),
- participants' perceptions of the effectiveness of the program, such as promotion of different psychosocial competencies, resilience, and overall personal development (16 items),
- the extent to which the participants would recommend the program to other people with similar needs (1 item),
- the extent to which the participants would join similar programs in the future (1 item),
- overall satisfaction with the program (1 item),
- things that the participants learned from the program (open-ended question),
- things that the participants appreciated most (open-ended question),
- opinion about the instructor(s) (open-ended question),
- areas that require improvement (open-ended question).

Similar to Form A, Form B includes the evaluation of the following:

- program implementers' perceptions of the program, such as program objectives, design, classroom atmosphere, interaction among the students, and the students' participation during class (10 items),
- program implementers' perceptions of their own practice, including their understanding of the course, teaching skills, professional attitude, involvement, and interaction with the students (10 items),
- program implementers' perceptions of the effectiveness of the program, such as promotion of different psychosocial competencies, resilience, and overall personal development of the students (16 items),
- the extent to which the workers would recommend the program to other students with similar needs (1 item),
- the extent to which the workers would teach similar programs in the future (1 item),
- overall satisfaction with the program (1 item),
- things that the workers obtained from the program (open-ended question),
- things that the workers appreciated most (open-ended question),
- difficulties encountered (open-ended question),
- areas that require improvement (open-ended question).

Based on the evaluation data collected in each school, program implementers in each school were required to complete a Tier 1 Program evaluation report where both quantitative and qualitative findings based on Forms A and B were summarized and described. In the last section of the report, the program implementers were requested to write down five conclusions regarding the program and its effectiveness. The involvement of the workers in writing the conclusions is consistent with the thesis that program implementers can give a more comprehensive and valid picture about the program quality and benefits to students. In addition, it is argued that they are proficient in accounting program effectiveness with reference to various aspects of the program, and providing recommendations for improving program arrangement and delivery in the real teaching context.

### 2.2. Data Analyses

In each cohort, the data generated from the five conclusions were analyzed using general qualitative analyses techniques [21] by two research assistants with a background in social work or psychology. The final coding and categorization were further cross-checked by another research colleague with a background in social work. All the research staff had received sufficient training on both quantitative and qualitative analyses. To guard against the subtle influence of such ideological biases and preoccupations of the coders, both intra- and interrater reliability on the coding were calculated. For intrarater reliability, each of the two research staff members who were primarily responsible for coding coded 20 randomly selected responses without looking at the original codes. For inter-rater reliability, another two research staff members who had not been involved in the data analyses coded the same 20 randomly selected responses independently without knowing the original codes given at the end of the scoring process. The data were also analyzed with reference to the principles of qualitative analyses proposed by Shek et al. [22].

In the previous analyses, the conclusions were categorized into several areas, including programs, implementers, benefits, difficulties, and recommendations. In the present secondary data analyses, the data in the existing datasets were also aggregated and analyzed with reference to these categories.

## 3. Results

Based on the 6,618 conclusions in the 1,327 evaluation reports, 14,390 meaningful units were extracted. Utilizing the analysis framework adopted in previous studies, these raw responses were further categorized into several categories, of which 28.75% related to views on the program (Table 2), 16.87% related to views on the program implementers (Table 3), 35.97% related to perceived effectiveness of the program (Table 4), 2.15% related to difficulties encountered during program implementation (Table 5), and 16.26% were recommendations (Table 6).

Regarding the conclusions related to the perceptions of the program, results in Table 2 showed that most of the responses were positive in nature. The percentage of positive responses in this domain was 80.22% on average. For the perceptions of the program implementers, findings in Table 3 also showed that a majority of the responses were positive in nature. Among the 2,427 responses, 97.33% were positive in nature. Findings on the perceived effectiveness of the program to the students are shown in Table 4, with a total of 5,176 meaningful units that could be categorized into several categories, including societal, familial, interpersonal, and personal enhancement. Overall, the positive effects of the program in different domains were evident, in which 95.78% were positive in nature.

To safeguard the reliability of the results, both the intra- and interreliability tests were conducted every year. The consolidated findings of the reliability analyses from 2005 to 2009 on stakeholders' perceptions toward program, instructors and program effectiveness can be seen in Table 7. The findings generally showed that the related figures were on the high side.

Despite the positive feedback, a small number of responses (*n* = 310, 2.15% of the total responses) were related to difficulties encountered. The difficulties included time constraints, difficulty in engaging the students, and inadequate school support (see Table 5). Lastly, suggestions for improvement can be seen in Table 6 (*n* = 2, 340; 16.26% of the total responses). It is noteworthy that some suggestions for improvement were contradictory (e.g., “deepen program content” versus “simplify and condense the program content” under the category of program content).

## 4. Discussion

Utilizing secondary data analyses, this study attempted to analyze the conclusions drawn by the program implementers of the Tier 1 Program of the Project P.A.T.H.S. in a series of studies over time. There are several unique characteristics of this study. First, a large number of reports (*n* = 1, 327) and schools (*n* = 244) were involved. Second, as there are very few published evaluation studies on positive youth development programs in different Chinese contexts, this is a pioneer addition to the literature. Third, as few subjective outcome evaluation studies are based on program implementers, the present study highlights the utility of including implementers' views in the evaluation of positive youth development programs. 

In line with previous findings based on the conclusions drawn by the program implementers [18–20], results showed that the majority of the responses related to the perceptions of the Tier 1 Program, instructors, and program effectiveness were positive in nature. These findings are consistent with the previous findings based on objective outcome evaluation, process evaluation, qualitative evaluation, and personal construct evaluation showing that the different stakeholders perceived the Tier 1 Program to be beneficial to the development of the program participants. In conjunction with the evaluation findings based on other methods, the picture that can be derived from the available evaluation findings is that the Project P.A.T.H.S. can promote the holistic development of young people in Hong Kong.

Despite the positive findings observed, difficulties encountered during program implementation and recommendations for improvement were noted, although the number of comments was low as compared to other areas. There are several areas of difficulties observed. First, consistent with previous studies, classroom discipline was one of the major hindrances to the implementation of the program because the program encourages active participation of the students. As Chinese teachers traditionally expect students to sit quietly and obediently in class, engaging students, yet maintaining class discipline, is a challenge. Another major hurdle observed was time management. For those schools where the program was implemented in the class teachers' periods, the time available for the class may not be adequate because class teachers usually have to deal with “class matters,” such as collection of class fees and discussion of class activities (e.g., classroom decoration during Christmas). Furthermore, program implementers are required to adopt a flexible and reflective approach in implementing the Tier 1 Program and they are expected to have much interaction with the students via structured activities, such as role play, group discussions, debates, and self-disclosure, which can arouse students' interest and motivation to learn. However, as Chinese teachers typically adopt an authoritarian rather than an egalitarian role in teaching, teachers might find it hard to play and share with the students. Finally, as Hong Kong is undergoing education reform, participation in the Project P.A.T.H.S. means intensive involvement of the workers, which may pose a challenge for the teachers and social workers. On the whole, the difficulties encountered and suggestions for improvement can serve as useful pointers to fine tune the program.

Regarding the evaluation methodology, the present study utilizes evaluation reports prepared by the program implementers via secondary data analyses. The approach to analyzing the evaluation reports prepared by the program implementers is consistent with several views in the evaluation literature. According to utilization-focused evaluation, involvement of the stakeholders in evaluation is an important element of evaluation [9]. In addition, there is a movement to treat teachers as researchers/evaluators, where teachers are treated as internal evaluators who carry out authentic assessment [23–27]. In the evaluation literature, the role of “internal evaluator” has been given increasing attention. Arguments supporting the involvement of stakeholders in evaluation can be seen in Shek and Ng [20].

Although the present findings can be interpreted as evidence supporting the merits and benefits of the Project P.A.T.H.S., several alternative explanations should be noted. The first alternative explanation is that the findings are due to insufficient evaluation expertise of the program implementers. Nevertheless, this alternative explanation can be partially dismissed because professional social workers and teachers received evaluation training in this project, and evaluation is also part of the training for social workers and teachers in Hong Kong. Furthermore, there are findings showing that subjective outcome evaluation converged with objective outcome evaluation findings [28]. The second alternative explanation is that the findings are due to biases, such as drawing positive conclusions for job retention. However, since the findings are consistent across time and across methods, this possibility is not high. Based on the principle of triangulation, an integration of the existing findings indicated that there is a consistent picture derived—the Tier 1 Program is beneficial to the development of the program participants. For example, with reference to the junior school years, evaluation findings showed that relative to control group students, students in the experimental schools generally had better holistic development and less problem behavior [29–31].

There are several limitations of the present integrative study based on nine databases. First, due to the nature of the secondary data analysis, it is not possible to have interaction with the program implementers. It would be helpful if some dialogues between the program implementers and researchers could be carried out in the future. Actually, the use of focus groups as an evaluation strategy has partially solved this problem. Second, because the conclusions written by the workers were not in great detail, the relevant findings failed to give us a thorough understanding of the implementation processes involved. Third, validity of the data derived from the present study relies on the assumption that program implementers can make reasonable and fair judgments about the program based on the subjective outcome evaluation findings. While this assumption might be met because teachers and social workers are trained to conduct practice evaluation in Hong Kong, inexperienced workers may have problems in integrating the subjective outcome evaluation findings and translating them into valid conclusions. Of course, it can be counterargued that systematic training before program implementation can reduce this problem to a great extent. Despite these limitations and in conjunction with the previous research findings described above, the findings in the present study provide further support for the effectiveness of the Tier 1 Program of the Project P.A.T.H.S. in Hong Kong.

## Figures and Tables

**Table 1:** Description of data characteristics from 2005 to 2009.

	S1				S2			S3	
	2005/06	2006/07	2007/08	2008/09	2006/07	2007/08	2008/09	2007/08	2008/09
	EIP	FIP	FIP	FIP	EIP	FIP	FIP	FIP	FIP
Total schools that	52	207	213	197	49	196	198	48	167
joined P.A.T.H.S									
(i) 10 h program	23	95	108	104	27	113	110	29	104
(ii) 20 h program	29	112	105	93	22	83	88	19	63
*Tier 1 Program*:									
Mean no. of sessions of program implementation	17.75	23.55	23.61	23.54	23.76	22.81	23.04	24.07	22.78
	(3–50)	(2–50)	(5–60)	(5–65)	(10–40)	(7–60)	(4–48)	(10–44)	(7–66)
No. of schools incorporated into formal curriculum	21	101	116	98	26	108	99	30	85
No. of schools incorporated into other modes	31	106	97	99	23	88	99	18	82
Mean no. of classes per school	4.58	4.66	4.69	4.56	4.51	4.62	4.64	4.56	4.67
	(2–7)	(1–8)	(1–8)	(1–8)	(1–7)	(1–8)	(1–8)	(1–8)	(1–8)
Total no. of instructors	419	1,582	1,630	1,458.5	336	1,486	1,473.5	344	1,186
Mean no. of teachers per school	5.13	5.47	5.63	5.75	2.27	5.59	5.63	2.25	5.40
	(0–17)	(0–14)	(0–28)	(0–28)	(0–6)	(0–15)	(0–20)	(0–6)	(0–20)
Mean no. social workers per school	2.63	2.13	2.00	1.75	4.55	1.97	1.76	4.90	1.68
	(0–8)	(0–9)	(0–8)	(0–10)	(0–12)	(0–8)	(0–10)	(0–12)	(0–7)
Total no. of students	8,679	35,735	36,343	31,280	8,167	33,449	33,583	7,708	28,157
Mean no. of students per school	166.90	172.63	171.05	158.78	166.67	170.66	169.61	160.58	168.60
	(37–240)	(17–280)	(16–267)	(5–251)	(32–240)	(12–280)	(15–263)	(26–240)	(28–240)
Total no. of student respondents	8,057	33,693	33,867	29,100	7,406	30,731	31,197	6,830	25,432
Mean no. of student respondents per school	154.94	162.77	159.00	147.72	151.14	156.80	157.56	142.29	152.29
	(37–212)	(15–265)	(14–267)	(3–251)	(32–220)	(12–243)	(15–263)	(23–213)	(22–229)
Total no. of instructor respondents	344	1,250	1,324	1,178	270	1,178	1,154	286	942
Mean no. of instructor respondents per school	6.62	6.04	6.22	5.98	5.51	6.01	5.83	5.96	5.64
	(1–21)	(1–18)	(1–29)	(1–24)	(2–15)	(1–17)	(1–16)	(1–18)	(1–16)

Note. S1: Secondary 1 level; S2: Secondary 2 level; S3: Secondary 3 level; EIP: Experimental Implementation Phase, FIP: Full Implementation Phase.

**Table 2:** Responses on views toward the program in different cohorts.

Grade	Year	*n*	Positive responses (%)	Top three responses among the positive responses
S1	2005/06	139	82%	Satisfied with the program (35)
				Interactive (10)
				Liked the program (6)
	2006/07	497	85%	Satisfied with the program (137)
				Positive impression towards the program (51)
				Clear objectives and strong theoretical support (39)
	2007/08	716	80%	Satisfied with the program (132)
				Positive impression towards the program (87)
				Good objectives/content/program design (67)
	2008/09	677	83%	Satisfied with the program (98)
				Positive impression towards the program (86)
				Good content/program design (46)
S2	2006/07	157	72%	Satisfied with the program (43)
				Like the program (10)
				Clear goals and objectives (8)
	2007/08	656	77%	Satisfied with the program (110)
				Positive impression towards the program (75)
				Clear objectives and strong theoretical support (46)
	2008/09	570	84%	Satisfied with the program (100)
				Positive impression towards the program (91)
				Liked the program (46)
S3	2007/08	153	74%	Satisfied with the program (46)
				Clear objectives and strong theoretical support (11)
				Positive impression toward the program (10)
	2008/09	572	85%	Satisfied with the program (86)
				Positive impression towards the program (82)
				Liked the program (46)

Total number of meaningful units		4,137		Average positive responses: 80.22%

**Table 3:** Responses on Views toward Program Implementers in Different Cohorts.

Grade	Year	*n*	Positive responses (%)	Top three responses among the positive responses
S1	2005/06	65	98%	Satisfied (42)
				Devoted (4)
				Very positive (3)
	2006/07	334	98%	Satisfied (110)
				Satisfactory performance (92)
				Commitment (21)
	2007/08	430	97%	Instructors were satisfied with their own performance (102)
				Satisfied (93)
				Instructor's attitude and performance influenced students' learning (45)
	2008/09	368	99%	Satisfied with/appreciated instructors' performance (119)
				Instructors were satisfied with their own performance (82)
				Sufficient preparation/understanding of the program (23)
S2	2006/07	84	99%	Good performance (35)
				Instructor satisfied with their own performance (27)
				Care about students (6)
	2007/08	354	97%	Satisfied (84)
				Instructors were satisfied with their own performance (80)
				Instructor's attitude and performance influenced students' learning (31)
	2008/09	428	94%	Satisfied with/appreciated instructors' performance (112)
				Instructors were satisfied with their own performance (96)
				Sufficient preparation/understanding of the program (30)
S3	2007/08	78	95%	Instructors were satisfied with their own performance (25)
				Students were satisfied with instructors' performance (22)
				Instructor's attitude and performance influenced students' learning (7)
	2008/09	286	99%	Satisfied with/appreciated instructors' performance (84)
				Instructors were satisfied with their own performance (56)
				Instructor's attitude and performance influenced students' learning (23)

Total number of meaningful units		2,427		Average positive responses: 97.33%

**Table 4:** Perceived effectiveness of the Tier 1 Program in different cohorts.

Grade	Year	*n*	Positive responses (%)	Top three responses
S1	2005/06	124	94%	Could help students' development (33)
				Promoted social ability (14)
				Promoted self-understanding (8)
				Enhanced self-confidence (8)
	2006/07	626	97%	Improved interpersonal relationship (69)
				Enhanced students' development (190)
				Enhanced self-understanding (45)
	2007/08	1032	94%	Promoted students' development (227)
				Promoted ability of differentiating between right and wrong (59)
				Could help instructors (48)
	2008/09	774	99%	Enhanced students' development (194)
				Promoted communication and interpersonal skills (77)
				Beneficial to students (59)
S2	2006/07	140	100%	Promoted students' development (41)
				Improved interpersonal relationship (16)
				Enhanced instructors and students relationship (10)
	2007/08	782	94%	Enhanced students' development (191)
				Promoted communication and interpersonal skill (68)
				Promoted ability of differentiating between right and wrong (37)
	2008/09	810	96%	Enhanced students' development (179)
				Beneficial to students (72)
				Promoted communication and interpersonal skills (59)
S3	2007/08	209	93%	Enhanced social skills (13)
				Enhanced students' development (49)
				Promoted ability of differentiating between right and wrong (12)
	2008/09	679	95%	Enhanced students' development (156)
				Promoted communication and interpersonal skills (53)
				Beneficial to students (41)

Total number of meaningful units		5,176		Average positive responses: 95.78%

**Table 5:** Difficulties highlighted by the respondents in different cohorts.

Grade	Year	*n*	Top three responses
S1	2005/06	21	Too much content led to overrun (5)
			Overlapping of content material (2)
			Time constraint (2)
	2006/07	62	Too much content (11)
			Time constraint (14)
			Could not match students' abilities/needs/interests (9)
			Difficult to maintain students' discipline while teaching (9)
	2007/08	28	Students' problem (7)
			School administration (5)
			Difficulties in classroom management (5)
	2008/09	55	Time constraints (18)
			Students' problem (9)
			Difficulties related to program content (6)
S2	2006/07	12	Too much content (3)
			Limited time (3)
			Students lack interest in the program (2)
	2007/08	25	Students' problem (10)
			School administration (4)
			High instructor-student ratio affected program effectiveness (2)
	2008/09	59	Time constraints (24)
			Had difficulties in preparing/implementing the lessons (9)
			Students' problem (7)
S3	2007/08	0	No difficulty was mentioned in this unit
	2008/09	48	Time constraints (21)
			Students' problem (6)
			Had difficulties in preparing/implementing the lessons (4)

Total number of meaningful units		310	

**Table 6:** Recommendations suggested by the program implementers in different cohorts.

Grade	Year	*n*	Top three items
S1	2005/06	75	Better match content and time (12)
			Interesting elements should be added (6)
			Need more diversified format (5)
	2006/07	336	Add more games/activities (30)
			Match content and time (28)
			Add interesting elements (26)
	2007/08	403	Add more games/activities (44)
			Content should be more lively, interesting, attractive, and innovative (31)
			Add more multimedia (29)
	2008/09	332	Content should be more lively, interesting, and attractive (41)
			Content should be adjusted to suit the needs, interests, and abilities of students (28)
			Add more games/activities (39)
S2	2006/07	84	More (diversified) activities and games (15)
			More lively and realistic (8)
			Modify the content (8)
	2007/08	352	Add more games/activities (46)
			Content should be more lively, interesting, and attractive (36)
			Content should be adjusted to suit the needs, interests, and abilities of students (19)
			Be more applicable to real-life situations (19)
			Need more diversified format (19)
	2008/09	368	Add more games/activities (43)
			Content should be more lively, interesting, and attractive (35)
			Be more applicable to real-life situations (33)
S3	2007/08	76	Add more games/activities (9)
			Content should be adjusted to suit the needs, interests, and abilities of students (8)
			Deepen program content (7)
	2008/09	314	Improve program content (30)
			Content should be more lively, interesting, and attractive (26)

Total number of meaningful units		2,340	Add more games/activities (26)

**Table 7:** Reliability results across cohorts.

Domain		Perceptions on views toward program		Perceptions on views toward implementers		Perceptions on perceived Effectiveness	
Grade	Year	Intrarater reliability	Interrater reliability	Intrarater reliability	Interrater reliability	Intrarater reliability	Interrater reliability
S1	2005/06	100%	83%	100%	83%	100%	93%
	2006/07	97.5%	95%	100%	97.5%	97.5%	97.5%
	2007/08	100%	95%	100%	100%	95%	95%
	2008/09	95%	90%	100%	100%	100%	100%
S2	2006/07	100%	100%	100%	100%	95%	100%
	2007/08	100%	100%	100%	100%	90%	100%
	2008/09	100%	95%	100%	100%	100%	95%
S3	2007/08	100%	100%	100%	100%	100%	100%
	2008/09	100%	90%	90%	90%	100%	95%

Average		99.17%	94.22%	98.89%	96.72%	97.50%	97.28%
